# Epidemiological Characteristics and Formation Mechanisms of Multidrug-Resistant Hypervirulent *Klebsiella pneumoniae*

**DOI:** 10.3389/fmicb.2020.581543

**Published:** 2020-11-20

**Authors:** Miran Tang, Xin Kong, Jingchen Hao, Jinbo Liu

**Affiliations:** ^1^Clinical Laboratory Department, Santai People’s Hospital, Mianyang, China; ^2^Department of Laboratory Medicine, Affiliated Hospital of Southwest Medical University, Luzhou, China

**Keywords:** *Klebsiella pneumoniae*, multi-drug resistance, hypervirulent, epidemiology, formation mechanism, plasmid, horizontal gene transfer, mobile genetic elements

## Abstract

Multi-drug resistance (MDR) and hypervirulence (hv) were exhibited by different well-separated *Klebsiella pneumoniae* lineages in the past, but their convergence clones—MDR-hypervirulent *K. pneumoniae* (HvKPs)—both highly pathogenic and resistant to most available antibiotics, have increasingly been reported. In light of the clonal lineages and molecular characteristics of the studied MDR-HvKP strains found in the literature since 2014, this review discusses the epidemiology of MDR-HvKPs, in particular summarizing the three general aspects of plasmids-associated mechanisms underlying the formation of MDR-HvKPs clones: MDR-classic *K. pneumoniae* (cKPs) acquiring hv plasmids, hvKPs obtaining MDR plasmids, and the acquisition of hybrid plasmids harboring virulence and resistance determinants. A deeper understanding of epidemiological characteristics and possible formation mechanisms of MDR-HvKPs is greatly needed for the proper surveillance and management of this potential threat.

## Introduction

*Klebsiella pneumoniae* is a clinically relevant opportunistic pathogen that causes a wide range of infections. Hypervirulent *K. pneumoniae* (hvKPs) and “classic” *K. pneumoniae* (cKPs) are two different variants of *K. pneumoniae* (Bialek-Davenet et al., 2014). The former are usually hypermucoviscous (HM) and are clinically characterized by their abilities to cause life-threatening invasive community-acquired infections, like entophthalmias and liver abscesses, in a healthy population (Shon et al., 2013). Fortunately, the majority of them have retained susceptibility to multiple commonly used antimicrobial agents except for ampicillin. The latter generally behave as opportunistic avirulent pathogens, causing nosocomial infections in hospitalized patients. Unfortunately, they have a propensity to acquire multiple resistant determinants, such as extended-spectrum β-lactamases (ESBLs) and/or carbapenemase, to be multi-drug resistant (MDR) strains making treatment more difficult (Navon-Venezia et al., 2017).

In the past, virulence and antibiotic-resistance have evolved separately in these two distinct *K. pneumoniae* clonal groups (Bialek-Davenet et al., 2014). However, in the face of antibiotic selection pressure, more and more isolates with combined hypervirulence (hv) and MDR have been detected and have reached public attention as “superbugs” with the possibility of causing untreatable invasive infections. To better understand such strains, this review focuses on summarizing and discussing the epidemiological characteristics and the possible formation mechanisms of them.

## Pathogen and Epidemiology

Over the past years, a “reference/standard” genotypic/phenotypic marker for hvKP has been lacking, and a positive string test indicating HM has been regarded as an important *in vitro* parameter for hvKP identification in some early studies (Yao et al., 2015). Yet, as research progressed, several controversies regarding the association of HM phenotype and hv have been raised. HM is not exclusive to hv, on the contrary, hvKP does not absolutely present HM (Carlos Catalan-Najera et al., 2017). In this review, studies involving hv (well-defined by virulence-related assays) or HM (positive for string test) strains were included.

*Klebsiella pneumoniae* has experienced the evolution of third-generation cephalosporin-, carbapenem-, and even polymyxin-resistance. Harboring a wide range of β-lactamases results in third-generation cephalosporin-resistance in *K. pneumoniae* isolates. Acquired resistance to carbapenems can be conferred by carbapenemase production, such as KPC, OXA, and MBLs which include NDM, VIM, IMP, as well as SIM (Tzouvelekis et al., 2012). Then, the prevalence of carbapenem-resistant bacteria has led to the use of polymyxins as a last-therapy option to treat associated infections, which has resulted in the emergence of polymyxins-resistant *K. pneumoniae.* This review includes literature about any kind of MDR phenotypes hvKPs.

We performed an exhaustive search of PubMed, MEDLINE, Web of Science, EMBASE, CNKI, and Wanfang database for English-language literature published before January, 2020, with the following search strategy (“hypervirulence”[All Fields] OR “hypervirulent”[All Fields] OR “hypermucoviscous”[All Fields]) AND (“*Klebsiella pneumoniae*”[MeSH Terms] OR “*Klebsiella pneumoniae*”[All Fields]) AND (“resistance”[All Fields] OR “resistant”[All Fields]), then summarized and classified these papers according to the country or region, STs, capsule types, plasmid replicon types, resistance loci, and formation mechanisms in Table 1, which we will refer to frequently throughout this section.

**TABLE 1:** Lists of papers referenced.

**Country**	**Sequence type(s)**	**Capsule type(s)**	**Resistance mechanism(s)**	**Plasmid replicon type(s)**	Formation mechanisms*	**References**
China	ST29 (*n* = 1)	K54 (*n* = 1)	*bla*_*NDM–*__5_ (*n* = 1)	p^*vir*^IncHI1/IncFIB p^*res*^IncX3	∘∘	Yuan et al., 2019
	ST1764 (*n* = 7) ST11 (*n* = 6)	K64 (*n* = 7) K47 (*n* = 4), K64 (*n* = 2)	*bla*_*NDM–*__1_ (*n* = 7) *bla*_*KPC–*__2_ (*n* = 6)	NA	NA	Liu Z. et al., 2019
	ST86 (*n* = 1)	K2 (*n* = 1)	*bla*_*NDM–1*_, *bla*_*KPC–2*_ co-carrying (*n* = 1)	p^*vir*^IncHI1/IncFIB p^*res*^IncFII(K) and p^*res*^IncN	∘∘	Liu Y. et al., 2019
	ST11 (*n* = 12) ST23 (*n* = 1) ST660 (*n* = 1) ST1660 (*n* = 1)	K64 (*n* = 7), K47 (*n* = 5) K1 (*n* = 1) K16 (*n* = 1) K1 (*n* = 1)	*bla*_*KPC–2*_ (*n* = 15)	NA	NA	Xu et al., 2019
	ST65 (*n* = 1)	K2 (*n* = 1)	*bla*_*CTX–M–*__3_, *bla*_*CTX–M–*__14_ coharboring	NA	NA	Fu et al., 2019
	ST23 (*n* = 1)	K1 (*n* = 1)	*bla*_*VIM–*__1_ (*n* = 1)	p^*vir*^IncHI1B/IncFIBk p^*res*^IncA and p^*res*^IncFII	∘∘	Dong et al., 2019
	ST23 (*n* = 1)	K1 (*n* = 1)	*bla*_*NDM–1*_ (*n* = 1)	NA	∘∘	Liu and Su, 2019
	ST25 (*n* = 16) ST11 (*n* = 3) ST375 (*n* = 1)	K2 (*n* = 16) Non-typeable (*n* = 3) K2 (*n* = 1)	*bla*_*KPC–2*_ (*n* = 10), *bla*_*NDM–1*_ (*n* = 1), ESBL_*S*_ (*n* = 5) *bla*_*KPC–2*_ (*n* = 3) ESBL_*S*_ (*n* = 1)	NA	NA	Li et al., 2019
	ST15 (*n* = 7)	KL112 (*n* = 7)	*bla*_*OXA–*__232_ (*n* = 7)	p^*res*–*OXA*^ColKP3-type p^*res*–*CTX*^ IncFII p^*res*–*MDR*^IncFIB p^*vir*^IncHI1B/IncFIB	∘∘	Shu et al., 2019
	ST23 (*n* = 1)	K1 (*n* = 1)	*bla*_*CTX–M–*__24_ (*n* = 1)	p^*vir*–*CTXM*^ IncHI1B/IncFIB	∘	Shen et al., 2019
	ST23 (*n* = 2) ST412 (*n* = 1) ST660 (*n* = 1) ST700 (*n* = 1)	K1 (*n* = 2) K57 (*n* = 1) K16 (*n* = 1) K1 (*n* = 1)	Undefined (*n* = 2) PhoQD150G (*n* = 1) PhoQD150G (*n* = 1) mcr-1 and PhoQD150G (*n* = 1)	NA	NA	Lu et al., 2018
	ST23 (*n* = 1)	K1 (*n* = 1)	*bla*_*DHA–*__1_ (*n* = 1)	p^*res*–*DHA*^IncHI5 p^*vir*^IncHI1B/IncFIB	∘∘	Xie et al., 2018
	ST2922 (*n* = 1)	K1 (*n* = 1)	*bla*_*DHA*_ and *bla*_*CTX–M–14*_ (*n* = 1)	p^*res*^IncR p^*vir*^IncFIB/IncHI1B	NA	Xu et al., 2018
	ST36 (*n* = 1)	K62 (*n* = 1)	*bla*_*KPC–2*_ (*n* = 1)	p^*ress*–*KPC*^ IncFII p^*vir*^ IncHI1/IncFIB	∘∘	Feng et al., 2018
	ST11 (*n* = 1)	K47 (*n* = 1)	*bla*_*KPC–2*_ (*n* = 1)	p^*ress*–*KPC*^IncR,IncFII, IncNp^*res*–*MDR*^ IncA/C2 p^*vir*^ IncHI1B/IncFIB	∘∘	Huang et al., 2018
	Unknown (*n* = 18)	K1 (*n* = 18)	ESBL_*S*_ and overexpression of efflux pumps (*n* = 18)	NA	NA	Lin et al., 2018
	ST86 (*n* = 1)	K2 (*n* = 1)	*bla*_*NDM–1*_- and *bla*_*KPC–2*_- Coproducing (*n* = 1)	NA	NA	Wei et al., 2018
	ST11 (*n* = 3)	Unknown (*n* = 3)	*bla*_*KPC–2*_ or *bla*_*NDM–1*_ (*n* = 3)	NA	NA	Wong et al., 2018
	ST11 (*n* = 1)	Unknown (*n* = 1)	tet(A) variant and *bla*_*KPC–2*_ (*n* = 1)	NA	∘	Gu et al., 2018
	ST11 (*n* = 3)	K47 (*n* = 3)	*bla*_*KPC–2*_ (*n* = 3)	NA	∘∘	Gu et al., 2017 Dong et al., 2018b
	ST11 (*n* = 4)	Unknown (*n* = 4)	tet(A) variant and *bla*_*KPC–2*_ (*n* = 1)	NA	∘∘	Yao et al., 2018
	ST11 (*n* = 16) ST268 (*n* = 2) ST65 (*n* = 1) ST692 (*n* = 1) ST595 (*n* = 1)	K20(*n* = 5),non-T(*n* = 11) K20(*n* = 1),non-T(*n* = 1)K2 (*n* = 1) non-T (*n* = 1) non-T (*n* = 1)	*bla*_*KPC–2*_ and *bla*SHV-11 (*n* = 16) *bla*_*KPC–2*_ and *bla*SHV-11 (*n* = 2) *bla*_*KPC–2*_ and *bla*SHV-11 (*n* = 1) *bla*_*KPC–2*_ and *bla*SHV-11 (*n* = 1) *bla*_*KPC–2*_ (*n* = 1)	NA	NA	Zhan et al., 2017
	ST23 (*n* = 2) ST268 (*n* = 3) ST65 (*n* = 1) ST17 (*n* = 1) ST420 (*n* = 1) ST367 (*n* = 1) ST1658 (*n* = 1) ST35 (*n* = 1)	K1 (*n* = 2) K1 (*n* = 1),K20 (*n* = 2) K2 (*n* = 1) Non-typeable (*n* = 1) K20 (*n* = 1) K1 (*n* = 1) K2 (*n* = 1) Non-typeable (*n* = 1)	SHV-75, CTXM-55, SHV-11, TEM-1, CTX-M-like, SHV-148, CTX-M-14, TEM-53 (*n* = 11)	NA	NA	Zhang Y.W. et al., 2016
	ST86 (*n* = 7) ST37 (*n* = 6) ST23 (*n* = 5)	Unknown	ESBL_*S*_ (*n* = 18)	NA	NA	Zhang J. et al., 2016
	ST661 (*n* = 1)	K1 (*n* = 1)	mcr-1 (*n* = 1)	NA	NA	Gu et al., 2016
	ST14 (*n* = 1)	K2 (*n* = 1)	*bla*_*NDM–5*_ (*n* = 1)	NA	NA	Liu et al., 2016
	ST11 (*n* = 1)	K1 (*n* = 1)	*bla*_*KPC–2*_ (*n* = 1)	p^*virs*–*KPC*^IncFIIk	∘	Wei et al., 2016
	ST23 (*n* = 1) ST23 (*n* = 1) ST1797 (*n* = 3)	K1 (*n* = 1) K1 (*n* = 1) K1 (*n* = 3)	*bla*_*KPC–2*_ (*n* = 1) *bla*_*KPC–2*_ (*n* = 1) *bla*_*KPC–2*_ (*n* = 3)	p^*virs*–*KPC*^IncHI1B/IncFIB NA NA	∘ ∘∘ ∘	Zhang R. et al., 2015 Dong et al., 2018a
	ST65 (*n* = 1)	K2 (*n* = 1)	SHV-11,TEM-53-producing ompK35,36 decreased (*n* = 1)	NA	NA	Zhang Y.W. et al., 2015
	ST25 (*n* = 2) ST65 (*n* = 5) ST11 (*n* = 1)	K2 (*n* = 2) K2 (*n* = 5) Non-typeable (*n* = 1)	*bla*_*KPC–2*_ (*n* = 6)	NA	NA	Yao et al., 2015
	Unknown (*n* = 5)	K1 and K2 (*n* = 5)	ESBL_*S*_ (*n* = 5)	NA	NA	Li et al., 2014
United Kingdom	ST101 (*n* = 3) ST383 (*n* = 3) ST147 (*n* = 4) ST15 (*n* = 2) ST48 (*n* = 1)	Unknown	*bla*OXA-48 and MDR (*n* = 1), MDR (*n* = 2) *bla*OXA-48 (*n* = 1), *bla*_*NDM–5*_ (*n* = 2), MDR (*n* = 1) *bla*_*NDM–1*_ and MDR (*n* = 2), MDR (*n* = 2) *bla*NDM and MDR (*n* = 1), MDR (*n* = 1) *bla*_*NDM–5*_ and MDR (*n* = 1)	p^*vir*–*MDR*–*OXA*–48^ and (or) -NDM IncFII(K)/IncFIB(K) p^*res*–*OXA*–48^IncL/M p^*res*–*NDM*–1^IncFIB(pQil) p^*vir*–*NDM*–5^ IncFIB(Mar)	∘	Turton et al., 2017, 2019
Argentina	ST25 (*n* = 1)	K2 (*n* = 1)	*bla*_*KPC–2*_ (*n* = 1)	NA	NA	Cejas et al., 2019
Japan	ST23 (*n* = 1)	K1 (*n* = 1)	*bla*IMP-6 (*n* = 1)qq	p^*res*–*IMP*–6^IncN	∘∘	Harada et al., 2019
Norway	ST15 (*n* = 2)	K24 (*n* = 2)	ESBL_*S*_ (*n* = 2)	p^*vir*–*CTXM*^ IncFIB_*K_1*_/IncFII_*K_1*_	∘	Lam et al., 2019
Iran	ST23 (*n* = 5)	K1 (*n* = 5)	*bla*VIM-2 (*n* = 5)	p^*res*–*VIM*–2^IncN	∘∘	Tabrizi et al., 2018
United Kingdom	ST23 (*n* = 1)	K1 (*n* = 1)	*bla*_*NDM–1*_ (*n* = 1)	NA	NA	Roulston et al., 2018
Italy	ST512 (*n* = 1)	Unknown (*n* = 1)	*bla*KPC-3 (*n* = 1)	NA	NA	Arena et al., 2017
France	ST86 (*n* = 1)	K2 (*n* = 1)	*bla*_*CTX–M–3*_ (*n* = 1)	p^*res*^IncL/M	NA	Surgers et al., 2016
India	ST2318 (*n* = 1)	Non-typeable (*n* = 1)	ESBL_*S*_ (*n* = 1)	NA	NA	Shankar et al., 2016b
India	ST11 (*n* = 1) ST43 (*n* = 1) ST231 (*n* = 1)	Unknown	*bla*_*OXA–232*_, *bla*_*OXA–*__181_, *bla*_*OXA–1*_, *bla*_*NDM–1*_	IncFIA IncFIB IncFII IncHI1B Col	NA	Shankar et al., 2016a
India	ST14 (*n* = 1)	Unknown (*n* = 1)	Mutation in OmpK36 (*n* = 1)	NA	NA	Rafiq et al., 2016
United States	ST23 (*n* = 1)	Unknown (*n* = 1)	*bla*_*KPC–2*_ (*n* = 1)	p^*res*–*KPC*^IncFIA	NA	Cejas et al., 2014
Brazil	ST11 (*n* = 7)	Unknown (*n* = 7)	*bla*_*KPC–2*_ qnrS1 *bla*CTX-M-2 (*n* = 7)	p^*ress*–*KPC*^IncFIIk	NA	Andrade et al., 2014

*Single ∘ represents acquiring hybrid plasmid by KPs, double ∘∘ represents acquiring additional plasmid(s), p^*vir*^ by MDR KPs or p^*res*^ by hvKPs, respectively.

From Table 1, we can see that MDR-HvKPs have mainly been detected since 2014 and have become research hotspot. All studies since 2014 involving both MDR and hv *K. pneumoniae* isolates account for a total of 47. China (including Taiwan) accounts for 33, and the remaining 13 are from other Asian countries (three from India, one from Japan, and one from Iran), Europe (two from the United Kingdom, one from France, one from Italy, and one from Norway), North America (one from United States), and South America (one from Brazil and another from Argentina). In our opinion, such geographical distribution is attributed to the prevalence characteristics of MDR and hv-KP strains around the world. For example, the high prevalence of both hvKPs and MDR-KPs in China and the significant proportions of incidence of MDR-KPs in other Asian countries (Lee et al., 2016, 2017) may contribute to the majority of reports about MDR-HvKPs coming from these regions.

By comparing the allelic sequences of seven housekeeping genes, multi-locus sequence typing (MLST) can structure *K. pneumoniae* populations into lineages, which are typically referenced by their sequence types (STs; e.g., ST11). The common MDR-KP strains are strongly linked to particular clonal complexes (CCs), like CC258, comprising ST258, ST11, ST512, ST340, ST437, etc. (Schweizer et al., 2019), CC15 and CC14, while hvKPs mainly belong to ST23 for the K1 capsular serotype and to ST86, ST65, and ST25 for K2 (Bialek-Davenet et al., 2014). Hence, the genetic backgrounds of the isolates, i.e., the strains stem from whether resistant or virulent lineage, can be determined by STs. MDR-HvKPs showed various STs in the literature (Table 1). Among them, the most prevalent ST was the KPC-producing CRKP highly related ST11, followed by the dominant hvKP lineage ST23. This was in accordance with the fact that ST11 and ST23 are predominant among MDR-KP and hvKP strains, respectively (Bialek-Davenet et al., 2014). In addition, STs showed different region distributions. In China, ST23 accounted for about 69.57% (16/23) in all hvKPs and ST11 took on almost the same high proportion 77.27% (51/66) in all MDR-KPs, respectively, which was consistent with the fact that both KPC-KP and hvKP are prevalent in China (Lee et al., 2016, 2017). While in other countries, except for India and Brazil, ST11 was almost undetected; instead, STs representing a virulent clone, such as ST23, ST25, and ST86, were more easily detected, which was in line with the fact that hvKP has increasingly prevailed in Europe and the Americas in recent years (Cubero et al., 2016; Lee et al., 2017).

## Possible Formation Mechanisms

Bacterial phenotypic changes including resistance and virulence acquisition are mainly driven by horizontal gene transfer (HGT) (in addition to chromosomal mutations) mediated by mobile genetic elements (MGEs), such as plasmids, insertion sequences (IS), transposons (Tn), integrons (In) and integrative conjugative elements (ICEs), driving dissemination and co-selection of virulence and resistance genes through genomic rearrangement during their replication or recombination process, mostly in Gram-negative pathogens including *K. pneumoniae* (Frost et al., 2005). Compared with transformation and transduction, thought to be secondary effects of other biological processes, conjugation plays a most impactful role in HGT due to the transfer of plasmids and ICEs *via* direct cell-to-cell contact (Pinilla-Redondo et al., 2018). Additionally, in most instances, hv or MDR phenotype selection genes transferred by Tn, In, and IS were mostly accumulated on plasmids followed by plasmid inter bacterial transfer. Consequently, as the most pivotal vectors for conjugation and genes recruitment, plasmids are arguably the most indispensable and essential elements throughout the entire convergence process of hv and MDR genes coming from strains of distinct backgrounds. Accordingly, in this section, we dissect the available mechanism studies apart from case reports (Table 1) to extract and further expand to three general aspects of plasmids-associated mechanisms underlying the convergence of hv and MDR phenotypes.

### MDR-cKPs Acquire Hypervirulence Plasmids

Virulence plasmid acquisition is an important mechanism for the increased virulence of MDR-cKPs. The best characterized virulence plasmids are the 224 kbp plasmid pK2044 from K1, ST23 strain NTUH-K2044; the 219 kbp plasmid pLVPK from K2, ST86 strain CG43; and the 121 kbp plasmid Kp52.145pII from K2, ST66 strain Kp52.145, on which the virulence-associated loci and genes were highly conservatively organized (Lam et al., 2018). There are some convincing and rational explanations to the comparative rarity of hv plasmids in avirulent MDR-KP clones compared to in hvKPs. First, dominant hv plasmids hosting KP ST23 accounts for only ≤ 2% of clinical *K. pneumoniae* isolates in the global range, except for in the Asia-Pacific rim, despite circulating among humans with hv plasmid maintenance for more than 100 years, far longer than the most well-known MDR clones (Wyres et al., 2020), suggesting that the hv plasmids rarely move horizontally and are highly restricted to hvKP clones, i.e., lineage specificity of plasmid distribution. Second, large numbers of plasmids, like hv plasmids in *K. pneumoniae*, devoid of genes permitting their transfer by conjugation and relying on the transfer function of other plasmids to enable their transfer, are mobilizable but not self-transmissible (Smillie et al., 2010), which radically limits the frequency of the horizontal transfer of hv plasmids. Third, as large low-copy-number plasmids, virulence plasmids contain specific replication and maintenance systems to ensure their transmission to daughter cells of specific genetic backgrounds (Million-Weaver and Camps, 2014), which is reminiscent of the fact that they might often impose fitness costs on other unsuitable host. Despite those restrictive factors, in fact, MDR-cKPs acquiring virulence plasmids have recently been reported. Yao et al. (2018) screened four ST11 CR-HvKP strains from clinical patients in Henan province, China, each of which carried both a KPC-2-encoding and a virulence plasmid. Further sequencing of the virulence plasmid showed high homology to pLVPK. Resembling that, Gu et al. (2018) reported the emergence of a tigecycline- and carbapenem-co-resistant ST11 hvKP isolate from a patient’s gut in Zhejiang, China. One of its three plasmids shared high homology with pLVPK and another co-carried *bla*_*KPC–*__2_ and *tet*(A). In the Lancet, Gu et al. reported a fatal outbreak of ST11 CR-HvKP strains in a Chinese hospital. In addition to their intrinsic conjugative MDR plasmids carrying *bla*_*KPC–*__2_ genes, the five representative causative strains acquired an additional virulence plasmid that aligned well to most parts of pLVPK (Gu et al., 2017). Subsequently, Dong et al. made the whole genome sequences (WGS) of three ST11 CR-HvKP isolates surveyed in the previous Gu et al.’s study and five plasmids harbored by each of them. The presence of homologous regions between the virulence plasmid and *bla*_*KPC–*__2_-bearing conjugative MDR plasmid suggested that their co-integrated transfer might mediate the transmission of the non-conjugative virulence plasmid from hvKP to ST11 CRKP (Dong et al., 2018b). The main explanation for these facts is that the virulence plasmids are not self-transmissible but often mobilized to access new hosts with the help of other ICEs or conjugative resistance plasmids encoding the conjugation transfer complex in the same host cell (Ramsay and Firth, 2017). Of note, the parental hvKPs, typically susceptible to antimicrobial agents, generally do not originally carry the conjugative resistance plasmid, so in this scenario, the event that hv plasmids are synergistically mobilized by conjugative resistance plasmids in the same host cell to new strains happens after the conjugative resistance plasmids are transferred into hvKPs first, as will be discussed in the next section.

### Hv (HM) KPs Obtain Multidrug-Resistance Plasmids

From the comparatively lower genome’s diversity in hvKPs than MDR-KPs, Wyres et al. (2019) inferred that hv clones are less likely to acquire resistance genes than MDR clones are to acquire virulence genes. Nevertheless, evidence seemly shows the opposite. We found that out of all the documents reviewed, the total number of Hv (HM) KP isolates obtaining MDR plasmids was much more than that of MDR-cKPs acquiring hv plasmids, 111 *vs.* 87, respectively. (Not absolutely, but it seems to be a doubt). In Zhang R. et al.’s (2015) study, both carbapenems-susceptible HvKP and CR-HvKP strains harbored an ∼200-kb virulence plasmid but the latter had acquired two additional plasmids with *bla*_*KPC–*__2_ gene located on a transferable plasmid. Tabrizi et al. described the emergence of VIM-2-encoding K1 ST23 CR-HvKP in an outbreak in Iran. Plasmid analysis revealed a class 1 In carrying *bla*_*VIM–*__2_ located on an ∼45-kb IncN conjugative plasmid (Tabrizi et al., 2018). As reported by Feng et al. (2018), a *bla*_*KPC–*__2_-mediated carbapenem-resistant ST36 hvKP clinical isolate had two plasmids, one IncHI1/IncFIB plasmid highly similar to the known pLVPK, another IncFII plasmid carrying *bla*_*KPC–*__2_ and proved self-transmissible. Similarly, the first IMP-producing K1 ST23 CR-HvKP in Japan carried a pLVPK-like plasmid and an IncN plasmid harboring class 1 In-mediated *bla*_*IMP–*__6_. According to the fact that *bla*_*IMP–*__6_ was, while *rmpA* was not, detected in the transconjugant, the authors inferred that the *bla*_*IMP–*__6_-carrying plasmid was conjugative, but the plasmid carrying virulence gene was not, and they were two individual plasmids (Harada et al., 2019). Liu et al. presented an NDM-1 and KPC-2 co-producing K2 ST86 CR-HvKP strain with four plasmids in China. Apart from an IncHI1/IncFIB virulence plasmid identical to pLVPK, the strain additionally acquired two carbapenemase-producing plasmids including *bla*_*NDM–*__1_-carrying IncN plasmid and IncFIIK plasmid which carried *bla*_*KPC–*__2_ and an array of other resistance elements (Liu Y. et al., 2019). Xie et al. delineated a *bla*_*DHA–*__1_-carrying IncHI5 plasmid which had a 26-kb accessory region where the *bla*_*DHA–*__1_ gene was located upstream of ISCR1 isolated from a K1 ST23 MDR-HvKP strain. Apart from this MDR plasmid, this strain carried another two plasmids, including its virulence plasmid (Xie et al., 2018). In Dong et al. (2019) study, a VIM-1-producing K1 ST23 CR-HvKP strain harbored three plasmids; a virulence plasmid highly homologous to that recovered from other ST23 hvKPs; and a *bla*_*VIM–*__1_-bearing plasmid possessing a unique resistance island structure presumably generated by multiple gene mobilization events. Yuan et al. (2019) showed that apart from an IncHI1/IncFIB pLVPK-like plasmid, a *bla*_*NDM–*__5_-carrying K54 ST29 CR-HvKP isolated from Sichuan, China, harbored a *bla*_*NDM–*__5_-carrying IncX3 self-transmissible plasmid. Recent WGS work of an NDM-1-producing K1 ST23 CR-HvKP in China, by Liu and Su (2019), showed that in addition to a pLVPK-like virulence plasmid, it had a conjugative resistance plasmid carrying a *bla*_*NDM–*__1_ and another six types of resistance genes surrounded by ISs. All of these studies were typical examples in which hvKP clones additionally acquired resistance plasmids all proved conjugative. On the theoretical basis that most large resistance plasmids encode their own transfer and are conjugative, we could further speculate from these papers that the surveyed phenotypically convergent strains originally carried a virulence plasmid and thereafter acquired extra resistance plasmids, which are readily transmitted by horizontal transfer between different lineages and species. Nevertheless, if we only consider the strong transfer of resistance plasmids, we cannot explain why the composite strains are still far less than MDR-KP strains. Therefore, we guess that the success of the MDR and hv convergence results from interaction of various positive and negative factors. Clear examples of the positive factors include the following: (i) most resistance plasmids encode all functions needed for their horizontal transfer, such as DNA replication and copy number control functions, mating pair formation genes, and an origin of transfer (oriT) (Pinilla-Redondo et al., 2018), which facilitated their transfer into other strains like hvKPs; (ii) acquisition of antibiotic resistance genes to become MDR-HvKPs promotes adaptive evolution of hvKP clones in an antibiotic environment; (iii) most acquisition of antibiotic resistance will reduce the virulence and fitness of the strain, especially in the absence of antibiotic selection (Durao et al., 2018); while in the era of antibiotics, antibiotic selection pressure promotes plasmids persistence once resistant mutants form; (iv) conjugative resistance plasmids are usually large and have a low-copy-number, which to some extent circumvent use of host material, and as such, has less effect on host fitness than high-copy-number ones; (v) host genetic background might be a pivotal determinant of plasmid fitness. Strain-dependent compensation to the cost of resistance acquisition might occur in hv strains. Intriguingly, not as many strains of concurrent hv and MDR phenotypes, as imagined in the context of so many positive advantages, implied the essential role of negative factors: (i) considerably lower genome diversity and plasticity than that of MDR-KP is a hint that there might be some sort of barrier for hvKP to uptake and/or integrate DNA fragments; (ii) hv strains typically sensitive to antibiotic are selectively killed by antimicrobial agents before they acquire resistance plasmids. Therefore, antibiotic usage creates conditions for the growth and development of MDR-cKP populations rather than producing a noticeable number of MDR-HvKPs. Altogether, we provide explanations for the complex interactions between resistance plasmids and hvKPs, which shed light on the mechanisms of Hv (HM) KPs obtaining and maintaining MDR plasmids. Notwithstanding Wyres et al.’s (2019) opinion posed at the beginning of this section, that hvKPs are unable or difficult to obtain resistance plasmids, it is possible to state that the genomic traits of hvKPs just weaken the strong transmission kinetics of MDR factors into themselves than into cKPs, producing relatively fewer MDR-hvKPs than MDR-cKPs. Under the interaction of many factors, it is still very easy for hvKPs to obtain resistance plasmids.

### Virulence-Resistance Hybrid Plasmids

**FIGURE 1:**
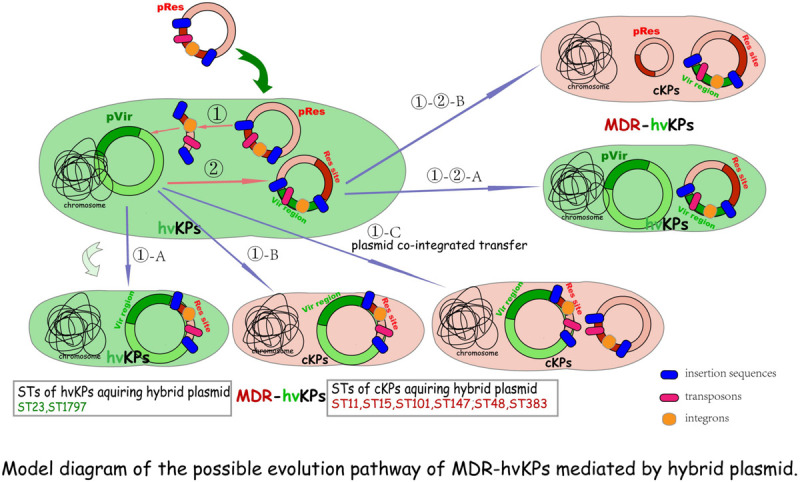
Model diagram of the possible evolution pathway of MDR-hvKPs mediated by a hybrid plasmid. **①** MDR plasmids first transfer into hvKPs, then antimicrobial resistance genes are integrated or transposed into hv plasmid harbored by hvKPs, resulting in the formation of hybrid plasmids with most hv genes-bearing regions and the MDR-hvKPs of hv-associated STs **(①-A)**. Alternatively, if the genes encoding the self-transfer conjugative system are integrated into the virulence plasmid, together with the resistance determinants, the hybrid plasmids will be conferred self-transmission and conjugativity transfer into any bacterial host including cKPs to become MDR-HvKPs **(①-B)**. In addition, with the help of other conjugative plasmids this hybrid plasmid can be possibly transferred into other cKPs to form MDR-hvKPs of MDR- or cKP-linked STs **(①-C)**. **②** If the In, Tn, and (or) Is further carry hv genes from the hv plasmid into other resistance plasmids, hybrid plasmids with most sites of resistance plasmid characteristics are formed. They can be transferred into either hvKPs **(①-②-A)** or cKPs **(①-②-B)** to form MDR-hvKPs via their conjugal transfer system.

Small MGEs, such as Tn, IS, or In, that “hitchhike” on the plasmids, especially resistance plasmids (Pinilla-Redondo et al., 2018), contribute to the capture and dissemination of MDR and/or hv genes whose co-existence on the same plasmid constitutes the perfect mosaic structure-hybrid plasmid. The transfer of the hybrid plasmids into either hvKP or cKP strains form MDR-HvKPs. The possible evolution pathway of MDR-HvKPs mediated by the hybrid plasmid is shown in model diagram Figure 1.

Some clear examples have been demonstrated. Zhang et al. reported the emergence of five K1 CR-HvKP strains causing fatal infections in hospital patients in Zhejiang Province, China. The K1 ST23 CR-HvKP70-2 harbored an ∼200-Kp plasmid on which *bla*_*KPC*_ and *rmpA* were located, and this plasmid was not transferred to *Escherichia coli*. Similarly, *bla*_*KPC–*__2_ was detected on two virulence genes-harboring plasmids which were not transferable to *E. coli* in other three genetically related K1 ST1797 isolates (Zhang R. et al., 2015). The non-conjugativity implied that these hv-MDR plasmids were very likely originally harbored by the hvKPs and carried the hv plasmid backbone, then formed mosaic structures *via* MGEs-mediated integration with the *bla*_*KPC–*__2_-bearing DNA fragment which comes from other conjugative *bla*_*KPC–*__2_-carrying resistance plasmid(s) transferred into the hv host before (Figure 1①-A). This hypothesis was subsequently proved in Dong et al.’s study where a hybrid plasmid recovered from CR-HvKP strain KP70-2 was found to be almost structurally identical to numerous known hv conferring plasmids harbored by other hvKP strains, except for an extra MDR-encoding region flanked by two copies of IS26 in the same orientation and MGEs-mediated resistance genes *dfrA14* and *bla*_*KPC–*__2_. The authors concluded that multiple IS elements were responsible for the integration of the MDR region into the virulence plasmid (Dong et al., 2018a). Similarly, the complete genome of an ESBL-producing K1 ST23 MDR-HvKP showed that the strain obtained a rare plasmid harboring virulence and *bla*_*CTX–M–*__24_ genes. Furthermore, a full-plasmid BLAST comparative analysis illustrated that this plasmid exhibited high similarity with three IncHI1B/IncFIB virulent plasmids retrieved from the GenBank, except for a unique *bla*_*CTX–M–*__24_-harboring region. Further exploration proved that following the IS-mediated *bla*_*CTX–M–*__24_ gene insertion, into the conserved virulence plasmid backbone region, the hybrid plasmid formed (Shen et al., 2019). In conclusion, these studies suggested that hv strains are capable of acquiring MDR determinants through the integration of the MDR region mediated by MGEs, like Tn, In, and seemly dominant IS, into its intrinsic virulence plasmid. The integration of additional resistance elements into the virulence plasmids of hvKPs constitutes perfect mosaic plasmids possessing dual characteristics of conserved virulent regions and newly acquired MDR-encoding sites, but maybe not conjugative (Figure 1①-A).

However, if the genes encoding a self-transfer conjugative system are integrated into the virulence plasmid together with the resistance determinants, the hybrid plasmids will be conferred self-transmission and conjugativity. Alternatively, with the help of other conjugative plasmids, this hybrid plasmid could possibly be mobilized to transfer. Hence, if the fitness cost brought into the host bacteria is not considered, they enable one-time simultaneous transfer of resistance and virulence genes into any type of *K. pneumoniae* clones, including cKPs, to facilitate emergence of MDR-HvKPs (Figures 1①-B,①-C). Indeed, a growing body of evidence supports the hypothesis. Huang et al. (2018) identified a hybrid virulent plasmid which comprised both parts of the pLVPK and an IncHI2-type resistance plasmid in a KPC-2-producing K47 ST11 MDR-HvKP strain in Taiwan. According to the study of Lam et al. (2019), both two ESBL-producing K24 ST15 *K. pneumoniae* isolates carried large hv-MDR mosaic plasmids which include sequences typical of IncFIB_*K*_ virulence plasmids, such as pK2044, fused with regions of homology with typical IncFIIK conjugative MDR plasmids. Similarly, Turton et al. (2019) also described hybrid plasmids containing both resistance and virulence clusters in 12 CR-HvKP isolates belonging to ST15/48/101/147/383. Yet, considering the special characteristics of classic hv plasmid, such as its notable lineage specificity, narrow-host-range, and low-copy-number traits, it might inhibit a second virulent plasmid transfer into the same strain, which thus might cause hvKPs to not acquire and maintain other hybrid plasmids with most hv genes-bearing regions. These surveyed CR-HvKP strains of hvKP-linked STs generally carrying a single hv or MDR-hv hybrid plasmid can support this hypothesis.

Besides, if the In, Tn, and/or Is further carry the virulent sites/genes from the hv plasmid into other resistance plasmids, hybrid plasmids with most sites of the resistance plasmid traits are formed. They can be transferred into either hvKPs (Figure 1①-②-A) or cKPs (Figure 1①-②-B) to form MDR-hvKPs mediated by their conjugal transfer system.

From a biological point of view, the highly mosaic nature of antimicrobial resistance and virulence determinants converging within a single vector, the purported co-selection, efficiently facilitates the evolution in two directions, which is a shortcut to an evolutionary success for a proficient bacteria, since selection by relevant antibiotics will also select for virulence traits (Turton et al., 2019). It should be noted that, due to the integration of multiple plasmids elements, including self-transfer conjugative system as well as expanded replicons number and host ranges, hybrid plasmids with MDR and hv biphenotypes can widely spread and infect many types of bacterial hosts and eventually become notorious environmental contaminators (Xie et al., 2020).

## Conclusion

In the context of what has already been reported by others regarding the MDR and hv convergence in *K. pneumoniae*, epidemiological characteristics and formation mechanisms of MDR-HvKPs researched in these papers have been discussed and elaborated in the current review.

Collectively, an epidemiology analysis enhances our understanding that the genetic background and geographical distribution characteristics of MDR-HvKP are highly consistent with the epidemic characteristics of hvKP and MDR strains and the monitoring and control of both will help prevent the occurrence of superbugs. The formation mechanism analysis has brought to light that hvKP and MDR strains could evolve into MDR-HvKPs through acquiring MDR, hv plasmids, or resistance/virulence hybrid plasmids. The formation mechanisms of biphenotypic composite bacteria are different in distinct genetic background *K. pneumoniae* clones, but the horizontal transfer of plasmids plays a decisive role. In addition, the adaptive evolution ability of the strain promotes the formation, persistence, and transmission of MDR-HvKP.

In the current review, we identified the important role of plasmid-mediated HGT endowing convergence of hv and MDR in *K. pneumoniae.* A plasmid-centered outlook, as opposed to the traditional host-centric view, should be taken into consideration by clinicians to turn the focal point from the specific host strains to resistance and virulence plasmids (and other MGEs) and to adopt surveillance strategies to track, hinder, or minimize the horizontal dissemination of them. Notably, avoiding drug abuse after the composite strains form may reduce the persistence of plasmids and extreme strains.

## Author Contributions

MRT and XK conceived, designed, and wrote the manuscript. JCH performed paper search and graphics visualization. JBL performed writing – reviewing and editing, supervision, and project administration. All authors read and approved the final manuscript.

## Conflict of Interest

The authors declare that the research was conducted in the absence of any commercial or financial relationships that could be construed as a potential conflict of interest.
